# Epigenetic reprogramming of cell identity: lessons from development for regenerative medicine

**DOI:** 10.1186/s13148-021-01131-4

**Published:** 2021-07-23

**Authors:** Amitava Basu, Vijay K. Tiwari

**Affiliations:** 1 Institute of Molecular Biology (IMB), 55128 Mainz, Germany; 2 Wellcome-Wolfson Institute for Experimental Medicine, School of Medicine, Dentistry and Biomedical Science, Queens University Belfast, Belfast, BT9 7BL UK

**Keywords:** Development, Epigenetic mechanisms, Transcription factors, Reprogramming, Regenerative medicine

## Abstract

Epigenetic mechanisms are known to define cell-type identity and function. Hence, reprogramming of one cell type into another essentially requires a rewiring of the underlying epigenome. Cellular reprogramming can convert somatic cells to induced pluripotent stem cells (iPSCs) that can be directed to differentiate to specific cell types. Trans-differentiation or direct reprogramming, on the other hand, involves the direct conversion of one cell type into another. In this review, we highlight how gene regulatory mechanisms identified to be critical for developmental processes were successfully used for cellular reprogramming of various cell types. We also discuss how the therapeutic use of the reprogrammed cells is beginning to revolutionize the field of regenerative medicine particularly in the repair and regeneration of damaged tissue and organs arising from pathological conditions or accidents. Lastly, we highlight some key challenges hindering the application of cellular reprogramming for therapeutic purposes.

## Background

Epigenetic mechanisms confer changes in the gene expression program without modulating the DNA sequence [1]. During mammalian development, the zygote undergoes a series of differentiation events to generate various cell types. The differentiation to various cell types requires the acquisition of cell-type-specific gene expression programs via epigenetic mechanisms [2–4]. These include DNA methylation, histone modifications, and noncoding RNAs such as micro-RNAs and long noncoding RNAs. The unique epigenetic landscape of each cell type determines its gene expression program that governs its identity and biological function [5, 6].

**Fig. 1:**
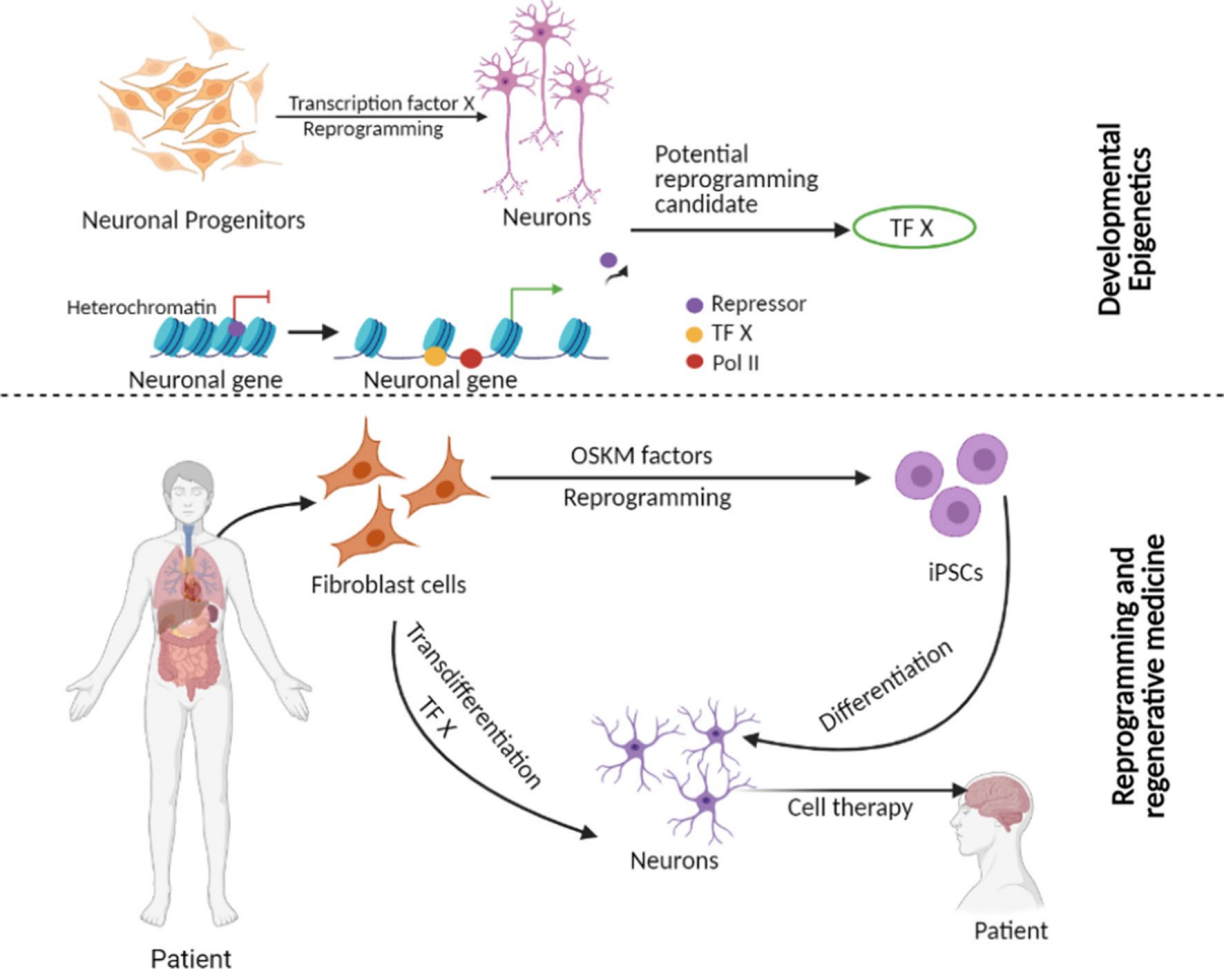
Scheme illustrating how knowledge of transcription factors and epigenetic mechanisms involved in developmental cell-fate decisions can guide efficient cellular reprogramming for therapeutic purposes. Created with https://biorender.com/

Over the years, numerous studies have attempted to convert differentiated cells into pluripotent cells or another cell type (direct reprogramming) using learnings from developmental biology (Fig. 1). The ultimate goal of generating the reprogrammed cell is to use them for regenerative medicine to restore structurally and functionally damaged tissues and organs. Currently, there are numerous clinical trials ongoing using reprogrammed cells and thus far have shown appreciable success. The reprogramming approaches include somatic cell nuclear transfer (SCNT), cell fusion, ectopic expression of specific transcription factors, micro-RNAs expression as well as by using small signaling molecules [7–10] (Table 1). It is becoming clear that such reprogramming involves remodeling of the epigenome eventually inducing a loss in molecular features of the original cell lineage and gain of new molecular features characteristic of the reprogrammed cell [11]:

**Table 1:** Summary of various successes in cellular reprogramming through ectopic expression of specific transcription factors or miRNAs, via CRISPR-Cas9 approach or via chemical inhibition of epigenetic machinery

Sl. no	Starting cell	Reprogrammed cell	Factors used	References
1	Fibroblast	Neurons	Ascl1, Brn2 and Myt1l	Vierbuchen et al. 2010 [26]
2	Fibroblast	Cardiomyocytes	Gata4, Mef2c and Tbx5	Ieda et al. 2010 [40]
3	Fibroblast	Hepatocytes	HNF1α, Foxa3 and Gata4	Huang et al. 2011 [44]
4	Fibroblast	iPSCs	Oct4, Klf4, Sox2 and cMyc	Yamanaka et al. 2006 [14]
5	Fibroblast	Myogenic cells	MyoD	Ito et al. 2017 [119]
6	Fibroblast	Neuron	miR-9/9* and miR-124	Yoo et al. 2011 [63]
7	Non-myocytes	Induced cardiomyocyte	miR-1, miR-133, miR-208 and miR-499	Jayawardena et al. 2012 [59]
8	B and T-cells	Macrophages	C/EBPα	Xie et al. 2004 [45]
9	ESCs	Trophoectodermal cells	Cdx2	Strumpf et al. 2005 [120]
10	Acinar cells	Insulin producing B cells	MafA, Pdx1 and Ngn3	Xu et al. 2013 [48]
11	Astrocytes	Glutamatergic Neurons	NeuroD1	Guo et al. 2014 [121]
12	mESC	Neurons	NeuroD1	Pataskar et. al. 2016 [29]
13	Neural precursor cell	Astrocyte	NFIA, ATF3 and RunX2	Tiwari et. al. 2018 [33]
14	Fibroblast	Oligodendrocyte	SOX10, ZFP536, OLIG2	Yang et al. 2013 [36]
15	Brain Pericytes	Neurons	Ascl1 and Sox2	Karow et. al. 2018 [122]
16	Pluripotent stem cell	Adipocyte	CEBPb, PRDM16	Ahfeldt et al. 2012 [123]
17	Fibroblast	Osteoblast	OCT4, RUNX2, OSX, MYC	Yamamoto et al. 2015 [124]
18	Fibroblast	iPSCs	CRISPR-dCas9 activation-OSKM and Lin28	Weltner et al. 2018 [67]
19	Fibroblast	Myoblast	CRISPR-dCcas9 activation of Myod enhancer	Liu et al. 2016 [68]
20	Neural progenitor cell	Neuron	CRISPR-dCcas9 activation of Sox1 promoter	Baumann et al. 2019 [69]
21	Fibroblasts	Neurons	CRISPR-dCcas9 activation of Brn2, Ascl1, and Myt1l	Black et al. 2016 [70]

### I. Ectopic expression of transcription factors

One of the most widely used methods for reprogramming cells is ectopic expression of transcription factors using adenovirus, lentivirus, retrovirus, etc., based transduction to deliver one or more transcription factors into primary cells. In stably reprogrammed cells, the epigenetic memory transmits across multiple cell divisions. The expression and activity of ectopically expressed transcription factors can alter the epigenetic state at the gene regulatory regions [12]. The presence of certain chromatin features has been shown to hinder the process of reprogramming of the cells, and hence, overcoming this barrier is an essential part of the reprogramming process [13]. We highlight below some examples where certain developmental transcription factors were used to reprogram cells and that function via epigenetic remodeling:

- The transcription factors Oct4, Sox2 and Klf4 are known to play a critical role in the pluripotency and differentiation potential of embryonic stem cells. A landmark study in the field was the reprogramming of the mouse fibroblast cells into embryonic stem cell-like iPSCs (induced pluripotent stem cells) using a cocktail of transcription factors Oct4, Sox2, Klf4, and cMyc (OSKM) [14]. During reprogramming, these factors cooperate with Polycomb repressive complex (PRC2) proteins to repress lineage-specific genes in the differentiated cells used for reprogramming to iPSCs [15, 16]. Such reprogramming events also involve loss of the repressive histone mark H3K27me3 [17–19]. Interestingly, during reprogramming, the mesenchymal-to-epithelial transition (MET) pathway is induced involving loss of mesenchymal marks including transcription factors such as Zeb1 and Snail1 and activation of epithelial markers like Cdh1, Epcam, etc. [20]. The OSKM factors can carry out loss of repressive methylation at promoter regions of pluripotency genes and a corresponding gain at the promoters of cell lineage-specific genes. The discovery of iPSCs has revolutionized the field of reprogramming, and several modifications to the original protocol have been made to generate better iPSCs and increase reprogramming efficiency [21–23]. There is numerous application of iPSC in regenerative medicine, some of which have been highlighted in the later section ‘Success stories of cellular reprogramming in regenerative medicine’.
- Ascl1 belongs to the basic helix-loop-helix (BHLH) family of transcription factors and was found to be essential for neuronal differentiation and functions via chromatin remodeling to generate neurons [24, 25]. The fibroblast cells can be converted directly to neurons with a cocktail of transcription factors Ascl1, Brn2 and Myt1l [26]. During reprogramming, Ascl1 triggers widespread chromatin accessibility in fibroblasts following Ascl1 overexpression and generates neurons [27]. The POU transcription factor Brn2 is known to be critical for neuronal differentiation during cortical development and is recruited via Ascl1 during reprogramming [28]. Myt1l is another established neuronal transcription factor essential for neurogenesis. Altogether, these three factors (BAM factors) induce rapid and efficient changes in the fibroblast transcriptome toward a neuronal one to enable successful reprogramming. The induced neurons generated from fibroblast have similar characteristics as cortical neurons with integration potential to the existing neuronal network and thus suitable for therapeutic use.
- The bHLH transcription factor NeuroD1 is induced during cortical development and was shown to remodel the chromatin landscape at target neuronal genes toward an active state to induce neuronal differentiation [29]. In a study, NeuroD1 could successfully convert mouse microglial cells directly into neurons [30]. Another study demonstrated that NeuroD1 can convert astrocytes to neurons using NeuroD1 [31]. Importantly, the neurons generated after reprogramming were successfully used in recovering the mouse brain with ischemic injury, clearly highlighting how the knowledge from development can be used for making a visible impact in regenerative medicine.
- NFIA was established as a gliogenic switch in the previous study [32, 33]. NFIA can bring about chromatin remodeling and demethylation of astrocyte-specific glial fibrillary acidic protein (GFAP) promoter to trigger this differentiation [32]. Our study has recently shown that at the onset of astrogliogenesis, NFIA binds to the target distal regulatory elements of critical astrocyte differentiation genes and converts primed to active chromatin to induce the required their expression [33]. Several studies have now shown that functional astrocytes can be generated via direct or indirect reprogramming using the transcription factor NFIA.
- Sox10 can regulate the expression of myelin protein and oligodendrocyte cell marker PDGFRα [34]. The bHLH transcription factor Olig2 is essential for oligodendrocyte development in collaboration with Nkx2.2. Zfp536 was shown to be induced late during oligodendrocyte differentiation [35]. Mouse fibroblasts can be converted to oligodendrocytes by expression of transcription factors Sox10, Olig2 and Zfp536 [36].
- The zinc finger transcription factor Gata4 is an established regulator of cardiac differentiation and regulates different cardiac-specific genes [37]. Mef2c is a mad box transcriptional factor and found to be a cofactor of Gata4 that regulates the cardiac muscle differentiation [38, 39]. Tbx5 is a member of the T-box transcription factor family, which activates genes involved in cardiomyocyte maturation. Fibroblast cells were directly reprogrammed into cardiomyocytes by overexpression of these three transcription factors Gata4, Mef2c, and Tbx5 [40]. The transdifferentiated cardiomyocytes are suitable for the treatment of damages from myocardial infarction in heart patients.
- Hepatocyte nuclear factor 1α (HNF1α) is important for the maintenance of hepatocytes [41, 42]. It is an activator of transcription and can regulate several genes during hepatogenesis. Loss of HNF1α function can cause fatty liver-related hepatocellular carcinoma. Foxa3 (hepatocyte nuclear factor 3 gamma) is a winged-helix transcription factor and helps maintain cellular glucose homeostasis [43]. A pioneering study showed how hepatocytes can be generated from fibroblasts by co-expression of HNF1α, Foxa3, and Gata4 [44].
- The differentiated B cells were successfully transdifferentiated to macrophages by the overexpression of C/EBPα and C/EBPβ [45]. These factors can inhibit the expression of Pax5 and consequently downregulate CD19.
- Pancreatic and duodenal homeobox 1 (Pdx1) is involved in the differentiation and maturation of β-cells [46]. Musculoaponeurotic fibrosarcoma oncogene homolog A (MafA) also plays an important role in preserving the function of the β-cells and an insulin activator in the cells. MafA can bind to the promoter region of the insulin gene and regulate its expression [47]. Neurogenin 3 (Ngn3) is required for islet-like cell production. In a well-recognized study, the pancreatic cells derived from the acinar cells were reprogrammed to insulin-producing cells by the expression of MafA, Pdx,1 and Ngn3 [48].
- The Tet family dioxygenases mediate sequential oxidation of 5-methylcytosine (5mC) into 5-hydroxymethylcytosine (5hmC), 5-formylcytosine (5fC) and 5-carboxylcytosine (5caC) [49]. The Tet proteins include Tet1, Tet2 and Tet3, which are involved in the process of epigenetic reprogramming of the cells [50, 51]. During the process of reprogramming 5hmC modification is increased and knockout of Tet proteins prevent reprogramming [52]. Tets are believed to reactivate Oct4 gene by demethylation of its promoter and enhancer regions and Tet1 can replace Oct4 in the OSKM reprogramming cocktail [46]. The iPSCs generated with Tet1, Sox2, Klf4, and c-Myc (TSKM) cocktail were found to be fully pluripotent. An interesting study highlighted how the Tet proteins can induce reprogramming by triggering mesenchymal-to-epithelial transition (MET) [53]. Tet3 was shown to regulate DNA methylation in the neural precursor cells and maintain the neural stem cell identity [54]. Knockdown of Tet3 causes upregulation of pluripotency genes in neural precursor cells. Further observations suggested that Tet3 is required for efficient reprogramming of fibroblasts into neurons. It was shown that knockout of all three Tets in MEFs can halt their reprogramming by preventing activation of micro-RNAs that are essential for MET during reprogramming [53]. Vitamin C, which was known to enhance reprogramming [55], was found to regulate Tet1-dependent 5hmC formation at loci involved in MET [56].

### II. micro-RNAs

miRNAs have been shown to exhibit the capacity to reprogram cells alone or in combination with other transcription factors. Owing to their relatively small size, miRNAs can be easily delivered in the cells to initiate reprogramming. Micro-RNAs such as the miR-302 is known to facilitate the reprogramming of human skin cells to iPSC-like cells [57, 58]. Furthermore, reprogramming of fibroblasts to cardiomyocytes is enhanced by using miR-1, miR-133, miR-208 and miR-499 [59]. miR-1 and miR-133 are known to inhibit cardiomyocyte proliferation and G1/S phase transition [60]. Furthermore, miR-208 induces the expression of cardiac transcription factors [61]. In addition, miR-499 functions as a regulator of cell proliferation during the late stages of cardiac differentiation [62]. It was also shown that fibroblasts can be reprogrammed to neurons using miR-9* and miR-124 which modulate the SWI/SNF-like BAF chromatin-remodeling complexes in neuronal progenitor cells [63]. Interestingly further, these miRNAs can work in synergy with the other transcription factor-like NeuroD2, Ascl1 and Mytl1 [63].

### III. CRISPR-Cas9-based genomic editing for reprogramming

Several recent studies have shown a successful application of Clustered Regularly Interspaced Short Palindromic Repeats (CRISPR) and catalytically inactive CRISPR-associated 9 (dCas9) nuclease for reprogramming of cells [64, 65]. This system is vastly robust and can be employed to correct disease-causing mutations or to repress or activate genes by targeting specific activators or repressors. A method to set up genome-wide reprogrammable transcriptional memory using CRISPR-based editing was recently reported, and it holds great potential for stable and specific editing of relevant genes for therapeutic purposes [66]. We present below some of the examples where CRISPR-dCas9 was successfully used for cellular reprogramming of cells using gene-specific targeting of selected epigenetic regulators.

**Fig. 2:**
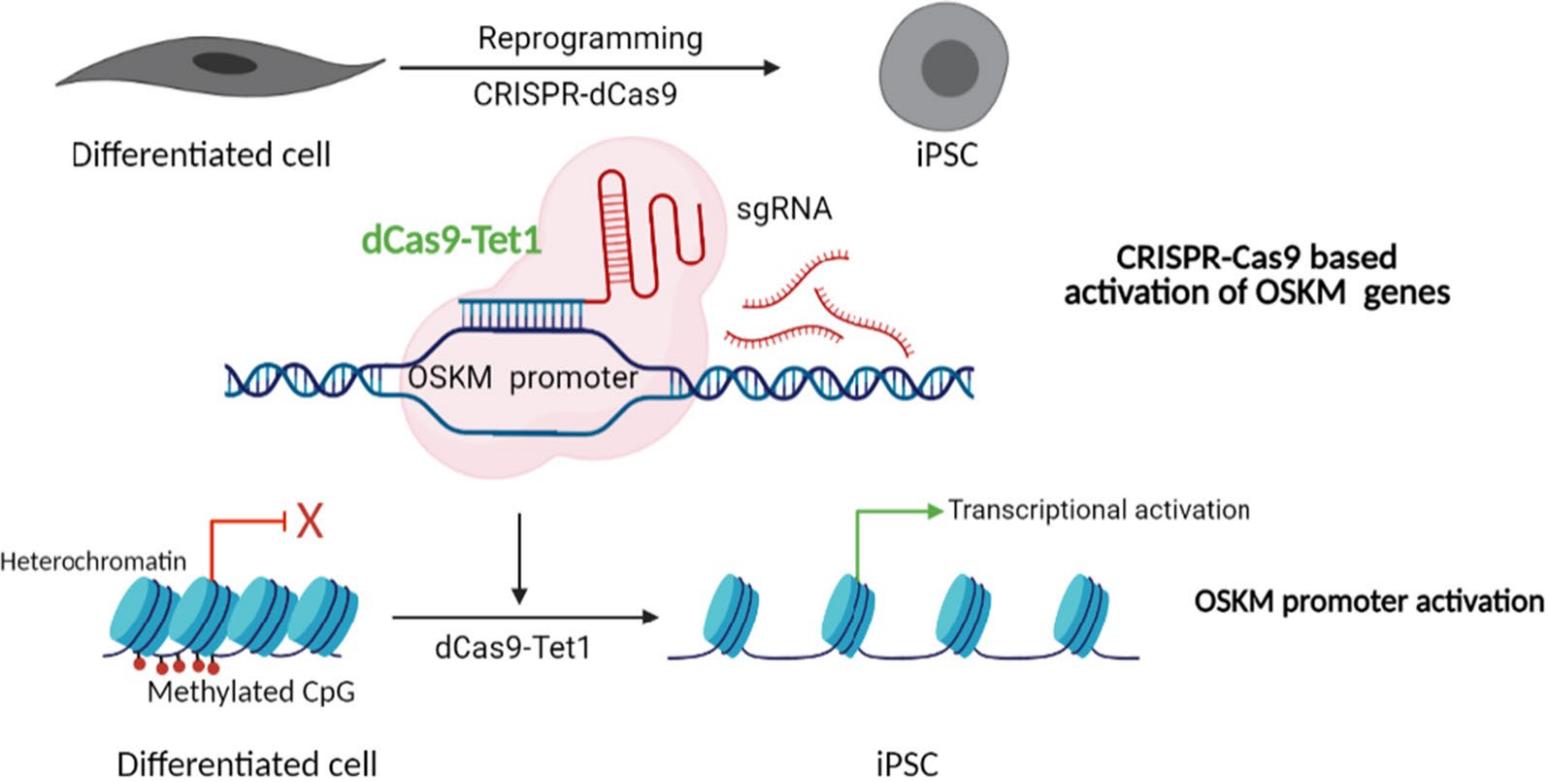
Scheme illustrating CRISPR-Cas9-mediated activation of endogenous OSKM genes for inducing pluripotent state from a differentiated cell type. Created with https://biorender.com/

- CRISPR-dCas9 was used successfully to activate the promoters of Oct4, Sox2, Klf4, Myc, and Lin28 genes to convert human fibroblast cells into iPSC cells [67] (Fig. 2). The reprogramming efficiency was further enhanced by targeting the Alu-motif embryonic genome activation genes.
- The CRISPR-dCas9 can be used to bring about targeted alteration of DNA methylation state to control gene expression of cell-fate genes and drive cell reprogramming. The DNA methyltransferase Dnmt3a or the DNA demethylase Tet1 can be fused to Cas9 to specifically target the regulatory elements of genes which should be epigenetically reprogrammed [68]. For example, the Tet1 fused Cas9 was used to activate the Myod enhancer and convert fibroblast into myoblast cells [68].
- CRISPR-dCas9 has also been used to enhance reprogramming efficiency. For example, scientists targeted the promoter of the Sox1 gene in the neural progenitor cells (NPC) with dCa9-Tet1 protein, resulting in increased expression of Sox1. This resulted in an enhancement in the differentiation potential of the NPCs where Sox1 acts as a master regulator [69].
- CRISPR-dCas9-based simultaneous induction of multiple promoters of Brn2, Ascl1, and Myt1l genes (BAM factors) could successfully convert mouse embryonic fibroblasts into neurons [70]. Such endogenous gene induction was rapid and stable over time and involved triggered chromatin remodeling at the target sites. This method offered better reprogramming efficiency to induced neurons as compared to the other transient transfection-based reprogramming.

### IV. Using chemical inhibitors for reprogramming

The field of reprogramming has greatly benefitted using small chemical molecules that have made a remarkable impact on increasing the efficiency as well as the scope of direct differentiation of cells. We will highlight a few examples below that have involved inhibition of epigenetic regulators:

- DNA methyltransferase inhibitors: The DNA methyltransferase inhibitor 5′-azacytidine (5′-azaC) can improve the reprogramming efficiency induced by OSKM in a dose-dependent manner [71]. A partially reprogrammed cell can also be driven into a fully reprogrammed cell by 5′-azaC treatment [72]. Another DNA methyltransferase inhibitor RG108 was shown to increase the reprogramming efficiency of Oct4 and Klf4 in the presence of BIX (G9a histone methyltransferase inhibitor) [73].
- Histone deacetylase inhibitors: HDAC inhibitor valproic acid (VPA) can induce reprogramming in the absence of cMyc overexpression. Furthermore, VPA improves the reprogramming efficiency with OSKM [71]. During the generation of OSKM-induced pluripotent stem cells (piPSCs) from MEFs, VPA can significantly increase the reprogramming efficiency [74]. Moreover, Two other HDAC inhibitors suberoylanilide hydroxamic acid (SAHA) and trichostatin A (TSA) were found to promote the MEF reprogramming efficiency [71]. Sodium butyrate, an HDAC inhibitor, can enhance the reprograming to human iPSC cells from adult or fetal fibroblast cells [75]. In addition, butyrate could induce the expression of certain pluripotency genes during reprogramming by catalyzing their promoter demethylation. Butyrate was suggested to be more efficient than VPA for Oct4 and Klf4-based reprogramming [76]. In another study, direct conversion of fibroblast cells into neurons was successfully carried out in the presence of VPA and some other inhibitors [77]. Moreover, the mouse fibroblasts can be directly reprogrammed into cardiomyocytes using a chemical cocktail including VPA [78]. Small molecules including VPA can also reprogram the astrocytes directly into neurons [79].
- Histone methyltransferase (HMT) inhibitors: BIX-01294, an HMT G9a inhibitor, can improve the reprogramming efficiency with Oct4/Klf4 in neural progenitor cells (NPCs) [73]. BIX is predicted to activate the Oct4 expression in the cells during reprogramming by inhibition of G9a-mediated H3K9me2 methylation.
- Histone demethylase inhibitor: Parnate is an LSD1 inhibitor, which in combination with CHIR99021(GSK-3 inhibitor) can reprogram the human primary keratinocytes into iPSCs upon overexpression of Oct4/Klf4 [80]. LSD1 inhibition with Parnate could partially convert the Epiblast stem cells (EpiSC) into pluripotent embryonic stem cell [81]. During this process, the expression of genes associated with the inner cell was found to be activated.

### Success stories of cellular reprogramming in regenerative medicine

The remarkable developments in the basic understanding and tools for reprograming have begun to show the clinical impact of cellular reprograming. The patient-derived cells have been successfully reprogrammed into different cell types and used for the treatment of underlying diseases. A few noticeable examples of such successful applications of reprogrammed cells for therapeutic use are highlighted below:

1. A Japanese woman was the first to receive cornea derived from iPSCs which significantly improved her vision [82]. The skin cells from a donor were reprogrammed into iPSCs, which were further differentiated into corneal cells. The use of such reprogrammed cornea can solve the problem associated with getting sufficient corneal tissue from the donor’s eye for transplantation.
2. Reprogrammed neuronal precursors were successfully implanted into a Parkinson’s disease patient in Japan [83]. The scientists used skin cells for reprogramming into iPSCs, which were differentiated into neuronal precursors that ultimately matured into dopamine-producing neurons. If successful, this treatment can be used to treat the tremors and walking issues in Parkinson's patients.
3. Cardiac tissues derived from reprogrammed iPSCs are currently under trials for use in patients with heart diseases [84]. The researchers plan to use the induced iPSCs to create sheets of heart muscle cells and grafted them into the heart. These sheets of heart cells can then produce growth factors that can help heal the damaged heart tissues in the adjoining regions.
4. Another potential application under testing involves the use of iPSCs generated precursor neurons to treat spinal cord injuries [85]. The precursor neuron cells could develop into neurons and glial cells when injected into the injured spinal cord in the monkey.
5. One of the earliest attempts in the treatment of a specific disease using iPSCs was for Duchenne Muscular Dystrophy (DMD), which results from mutations in the dystrophin gene that leads to muscular degeneration and ultimately loss of movement [41]. Here the approach involved converting the pluripotent stem cells into muscle cells by activation of MyoD. MyoD is a basic helix-loop-helix regulatory factor and responsible for the expression of muscle-specific genes in the embryo. Specific manipulation of epigenetic circuitry with HDACi is suggested to play a vital role in this targeted differentiation [86]. Transplantation of these transformed myocytes into adults suffering from DMD is expected to improve their condition by muscle regeneration [87].

### Challenges in the field

Despite the revolutionary potential of reprogramming for therapeutics, several issues have created obstacles for a successful use of reprogrammed cells for therapeutic purposes. Some of these issues are highlighted below-

- Incomplete resetting of epigenetic mark

During the process of reprogramming of cells, resetting of epigenetic marks such as DNA methylation is not complete [88]. This can lead to considerable differences between the individual reprogrammed cells and affect the differentiation potential and suitability of such cells for therapeutic purposes. In addition, such partially reprogrammed cells have higher tendency to become tumorigenic. The incomplete reprogramming can also lead to persistence of founder cell traits, which is not suitable for therapeutics.

b) Mutagenesis due to retroviruses

Many reprogramming protocols require retroviruses to deliver reprogramming factors into cells. These retroviruses can cause insertional mutagenesis in reprogrammed cells [89]. The integration of retrovirus can also lead to activation of retrotransposable elements in cells. To overcome these problems, there is a shift toward methods of reprogramming independent of retroviruses such as chemical-induced reprogramming and use of episomal vectors [90, 91].

c) Neoplastic development

The genes used to trigger the process of reprogramming such as OSKM can lead to neoplastic development in reprogrammed cells by getting activated during a later time point. This calls for the development of alternate approaches for reprogramming to minimize the carcinogenic potential of reprogrammed cells [92–94]. Tumors can also be initiated by the disruption of tumor suppression genes or the action of oncogenes during genomic integrations mediated by virus used for reprogramming.

d) Immunogenic incompatibility

In case the transplanted reprogrammed cell is derived from cells other than the patient itself, there is the possibility of immunogenic reaction in the receiver patient. The immune reaction elicited by such cells can decrease the survival of transplanted reprogrammed cells. In such cases, the patient is prescribed lifelong immunosuppressants which in turn can increase the susceptibility of the patient to certain opportunistic infections and other health complications [95, 96].

## Conclusions

The generation of iPSCs or specific transdifferentiated cells has created a new paradigm in the field of regenerative medicine with a wide range of applications including understanding the fundamental biology of cell specification, to drug screening to the treatment of patients [97–99]. The derived iPSCs can be used either for in vitro culturing for screening various drugs to treat the disease or for cell replacement therapy for the treatment of underlying diseases [100]. In patients suffering from diseases such as Parkinson’s disease, patient-derived iPSCs are generated and underlying mutations corrected by gene therapy and subsequently differentiated into specific neurons [101]. These reprogrammed cells can be transplanted back to the patient for therapy. Similar approaches for diseases such as muscular dystrophy, Down syndrome, Fanconi anemia and Huntington’s disease are under trial by various laboratories [102–105]. The use of patient-specific reprogrammed cells can circumvent various risks associated with the rejection of transplanted cells in the body as well as be a source of unlimited cells for therapy. In addition, the ability to study disease in a Petri plate using the iPSCs derived from the patient offers a unique opportunity to study the diseased phenotype for its better treatment. It would be vital to decipher the epigenetic mechanisms underlying these processes comprehensively and further optimize the protocol for the generation of iPSCs or transdifferentiated cells from patient cells. Exciting new approaches like CRISPR-cas9-based activation of transcription factors as well as computational modeling to screen large number of transcription factors for reprogramming ability offer an excellent opportunity to investigate the role of more than 2000 transcription factors for reprogramming [67, 106, 107]. Recently one of the focuses in regenerative therapeutics has been toward directed reprogramming of one cell type into another by transdifferentiation without the need to go through the intermediate pluripotent cell stage [108–110]. Transdifferentiated cells can be generated at better efficiency and in a shorter interval of time compared to iPSC cells. Another huge advantage with transdifferentiation is that the cells can be reprogrammed directly in the affected tissue or organ without the need to derive pluripotent cells outside the body of the organism. Certain signaling molecules including growth factors present in the microenvironment of the transdifferentiated cells can enhance the transdifferentiation potential of the cell in vivo [111, 112].

Another important aspect for clinical application of these cells is regarding the safety including long-term behavior of these cells and tumorigenic potential once they are transplanted back into the patients [113, 114]. There have also been efforts to generate the iPSCs without viral genome integration or even without the use of viruses for delivery of the transcription factors in the cell as the integration of viral genome in recipient cell is associated with tumorigenic consequences [115]. The aim therefore should be to generate homogeneous reprogrammed cells that resemble the naturally occurring cell for therapeutics. A combinatorial approach using small chemical and transcription factors might pave the way for better-reprogrammed cells with increased reprogramming efficiency that might be a game-changer in the field of therapeutics [116]. The reprogrammed cells need to be mature as well as retain the ability to retain the reprogrammed memory across cell divisions [117]. The delivery of reprogrammed cells into the body can be critical depending on the target area of the body [118]. The successful application of reprogrammed cells in therapeutics is thus dependent on overcoming all these hurdles before we can apply this technique for the treatment of a wide range of diseases.

## Data Availability

There are no data and material associated with this review.

## Abbreviations

iPSCs: Induced pluripotent stem cells
EpiSC: Epiblast stem cells
SCNT: Somatic cell nuclear transfer
PRC2: Polycomb repressive complex
CRISPR: Clustered regularly interspaced short palindromic repeats
Cas9: CRISPR-associated 9 ()
NPC: Neural progenitor cells
5′-azaC: 5′-Azacytidine
DMD: Duchenne muscular dystrophy

## References

[1] Waddington CH, Kacser H. The strategy of the genes: a discussion of some aspects of theoretical biology: Allen & Unwin; 1957.

[2] Jaenisch R, Bird A (2003). Epigenetic regulation of gene expression: how the genome integrates intrinsic and environmental signals. Nat Genet.

[3] Li E (2002). Chromatin modification and epigenetic reprogramming in mammalian development. Nat Rev Genet.

[4] Gibney ER, Nolan CM (2010). Epigenetics and gene expression. Heredity (Edinb).

[5] Goldberg AD, Allis CD, Bernstein E (2007). Epigenetics: a landscape takes shape. Cell.

[6] Dean W, Santos F, Reik W (2003). Epigenetic reprogramming in early mammalian development and following somatic nuclear transfer. Semin Cell Dev Biol.

[7] Hou P, Li Y, Zhang X, Liu C, Guan J, Li H (2013). Pluripotent stem cells induced from mouse somatic cells by small-molecule compounds. Science.

[8] Matoba S, Zhang Y (2018). Somatic cell nuclear transfer reprogramming: mechanisms and applications. Cell Stem Cell.

[9] Sanges D, Lluis F, Cosma MP (2011). Cell-fusion-mediated reprogramming: pluripotency or transdifferentiation? Implications for regenerative medicine. Adv Exp Med Biol.

[10] Takahashi K, Yamanaka S (2016). A decade of transcription factor-mediated reprogramming to pluripotency. Nat Rev Mol Cell Biol.

[11] Watanabe A, Yamada Y, Yamanaka S (2013). Epigenetic regulation in pluripotent stem cells: a key to breaking the epigenetic barrier. Philos Trans R Soc Lond B Biol Sci.

[12] Simonsson S, Gurdon J (2004). DNA demethylation is necessary for the epigenetic reprogramming of somatic cell nuclei. Nat Cell Biol.

[13] Gaspar-Maia A, Alajem A, Meshorer E, Ramalho-Santos M (2011). Open chromatin in pluripotency and reprogramming. Nat Rev Mol Cell Biol.

[14] Takahashi K, Yamanaka S (2006). Induction of pluripotent stem cells from mouse embryonic and adult fibroblast cultures by defined factors. Cell.

[15] Pereira CF, Piccolo FM, Tsubouchi T, Sauer S, Ryan NK, Bruno L (2010). ESCs require PRC2 to direct the successful reprogramming of differentiated cells toward pluripotency. Cell Stem Cell.

[16] Fragola G, Germain P-L, Laise P, Cuomo A, Blasimme A, Gross F (2013). Cell reprogramming requires silencing of a core subset of polycomb targets. PLoS Genet.

[17] Onder TT, Kara N, Cherry A, Sinha AU, Zhu N, Bernt KM (2012). Chromatin-modifying enzymes as modulators of reprogramming. Nature.

[18] Mansour AA, Gafni O, Weinberger L, Zviran A, Ayyash M, Rais Y (2012). The H3K27 demethylase Utx regulates somatic and germ cell epigenetic reprogramming. Nature.

[19] Rao RA, Dhele N, Cheemadan S, Ketkar A, Jayandharan GR, Palakodeti D, Rampalli S (2015). Ezh2 mediated H3K27me3 activity facilitates somatic transition during human pluripotent reprogramming. Sci Rep.

[20] Li R, Liang J, Ni S, Zhou T, Qing X, Li H (2010). A mesenchymal-to-epithelial transition initiates and is required for the nuclear reprogramming of mouse fibroblasts. Cell Stem Cell.

[21] Yamanaka S, Blau HM (2010). Nuclear reprogramming to a pluripotent state by three approaches. Nature.

[22] Polo JM, Anderssen E, Walsh RM, Schwarz BA, Nefzger CM, Lim SM (2012). A molecular roadmap of reprogramming somatic cells into iPS cells. Cell.

[23] Hochedlinger K, Jaenisch R (2015). Induced pluripotency and epigenetic reprogramming. Cold Spring Harb Perspect Biol.

[24] Jorstad NL, Wilken MS, Todd L, Finkbeiner C, Nakamura P, Radulovich N (2020). STAT signaling modifies Ascl1 chromatin binding and limits neural regeneration from muller glia in adult mouse retina. Cell Rep.

[25] Castro DS, Martynoga B, Parras C, Ramesh V, Pacary E, Johnston C (2011). A novel function of the proneural factor Ascl1 in progenitor proliferation identified by genome-wide characterization of its targets. Genes Dev.

[26] Vierbuchen T, Ostermeier A, Pang ZP, Kokubu Y, Südhof TC, Wernig M (2010). Direct conversion of fibroblasts to functional neurons by defined factors. Nature.

[27] Wapinski OL, Lee QY, Chen AC, Li R, Corces MR, Ang CE (2017). Rapid chromatin switch in the direct reprogramming of fibroblasts to neurons. Cell Rep.

[28] Wapinski OL, Vierbuchen T, Qu K, Lee QY, Chanda S, Fuentes DR (2013). Hierarchical mechanisms for direct reprogramming of fibroblasts to neurons. Cell.

[29] Pataskar A, Jung J, Smialowski P, Noack F, Calegari F, Straub T, Tiwari VK (2016). NeuroD1 reprograms chromatin and transcription factor landscapes to induce the neuronal program. EMBO J.

[30] Matsuda T, Irie T, Katsurabayashi S, Hayashi Y, Nagai T, Hamazaki N (2019). Pioneer factor NeuroD1 rearranges transcriptional and epigenetic profiles to execute microglia-neuron conversion. Neuron.

[31] Chen Y-C, Ma N-X, Pei Z-F, Wu Z, Do-Monte FH, Keefe S (2020). A NeuroD1 AAV-based gene therapy for functional brain repair after ischemic injury through in vivo astrocyte-to-neuron conversion. Mol Ther.

[32] Tchieu J, Calder EL, Guttikonda SR, Gutzwiller EM, Aromolaran KA, Steinbeck JA (2019). NFIA is a gliogenic switch enabling rapid derivation of functional human astrocytes from pluripotent stem cells. Nat Biotechnol.

[33] Tiwari N, Pataskar A, Péron S, Thakurela S, Sahu SK, Figueres-Oñate M (2018). Stage-specific transcription factors drive astrogliogenesis by remodeling gene regulatory landscapes. Cell Stem Cell.

[34] Finzsch M, Stolt CC, Lommes P, Wegner M (2008). Sox9 and Sox10 influence survival and migration of oligodendrocyte precursors in the spinal cord by regulating PDGF receptor alpha expression. Development.

[35] Dugas JC, Ibrahim A, Barres BA (2007). A crucial role for p57(Kip2) in the intracellular timer that controls oligodendrocyte differentiation. J Neurosci.

[36] Yang N, Zuchero JB, Ahlenius H, Marro S, Ng YH, Vierbuchen T (2013). Generation of oligodendroglial cells by direct lineage conversion. Nat Biotechnol.

[37] Yilbas AE, Hamilton A, Wang Y, Mach H, Lacroix N, Davis DR (2014). Activation of GATA4 gene expression at the early stage of cardiac specification. Front Chem.

[38] Wang L, Liu Z, Yin C, Asfour H, Chen O, Li Y (2015). Stoichiometry of Gata4, Mef2c, and Tbx5 influences the efficiency and quality of induced cardiac myocyte reprogramming. Circ Res.

[39] Chen JX, Krane M, Deutsch M-A, Wang L, Rav-Acha M, Gregoire S (2012). Inefficient reprogramming of fibroblasts into cardiomyocytes using Gata4, Mef2c, and Tbx5. Circ Res.

[40] Ieda M, Fu J-D, Delgado-Olguin P, Vedantham V, Hayashi Y, Bruneau BG, Srivastava D (2010). Direct reprogramming of fibroblasts into functional cardiomyocytes by defined factors. Cell.

[41] Shih DQ, Bussen M, Sehayek E, Ananthanarayanan M, Shneider BL, Suchy FJ (2001). Hepatocyte nuclear factor-1alpha is an essential regulator of bile acid and plasma cholesterol metabolism. Nat Genet.

[42] Odom DT, Zizlsperger N, Gordon DB, Bell GW, Rinaldi NJ, Murray HL (2004). Control of pancreas and liver gene expression by HNF transcription factors. Science.

[43] Shen W, Scearce LM, Brestelli JE, Sund NJ, Kaestner KH (2001). Foxa3 (hepatocyte nuclear factor 3gamma ) is required for the regulation of hepatic GLUT2 expression and the maintenance of glucose homeostasis during a prolonged fast. J Biol Chem.

[44] Huang P, He Z, Ji S, Sun H, Xiang D, Liu C (2011). Induction of functional hepatocyte-like cells from mouse fibroblasts by defined factors. Nature.

[45] Xie H, Ye M, Feng R, Graf T (2004). Stepwise reprogramming of B Cells Into Macrophages. Cell.

[46] Gao T, McKenna B, Li C, Reichert M, Nguyen J, Singh T (2014). Pdx1 maintains β cell identity and function by repressing an α cell program. Cell Metab.

[47] Ye DZ, Tai M-H, Linning KD, Szabo C, Olson LK (2006). MafA expression and insulin promoter activity are induced by nicotinamide and related compounds in INS-1 pancreatic beta-cells. Diabetes.

[48] Xu H, Tsang KS, Chan JCN, Yuan P, Fan R, Kaneto H, Xu G (2013). The combined expression of Pdx1 and MafA with either Ngn3 or NeuroD improves the differentiation efficiency of mouse embryonic stem cells into insulin-producing cells. Cell Transplant.

[49] Pastor WA, Aravind L, Rao A (2013). TETonic shift: biological roles of TET proteins in DNA demethylation and transcription. Nat Rev Mol Cell Biol.

[50] Epigenetics SH (2014). Reprogramming with TET. Nat Rev Genet.

[51] Piccolo FM, Bagci H, Brown KE, Landeira D, Soza-Ried J, Feytout A (2013). Different roles for Tet1 and Tet2 proteins in reprogramming-mediated erasure of imprints induced by EGC fusion. Mol Cell.

[52] Dawlaty MM, Breiling A, Le T, Barrasa MI, Raddatz G, Gao Q (2014). Loss of Tet enzymes compromises proper differentiation of embryonic stem cells. Dev Cell.

[53] Hu X, Zhang L, Mao S-Q, Li Z, Chen J, Zhang R-R (2014). Tet and TDG mediate DNA demethylation essential for mesenchymal-to-epithelial transition in somatic cell reprogramming. Cell Stem Cell.

[54] Santiago M, Antunes C, Guedes M, Iacovino M, Kyba M, Reik W (2020). Tet3 regulates cellular identity and DNA methylation in neural progenitor cells. Cell Mol Life Sci.

[55] Esteban MA, Wang T, Qin B, Yang J, Qin D, Cai J (2010). Vitamin C enhances the generation of mouse and human induced pluripotent stem cells. Cell Stem Cell.

[56] Chen J, Guo L, Zhang L, Wu H, Yang J, Liu H (2013). Vitamin C modulates TET1 function during somatic cell reprogramming. Nat Genet.

[57] Anokye-Danso F, Trivedi CM, Juhr D, Gupta M, Cui Z, Tian Y (2011). Highly efficient miRNA-mediated reprogramming of mouse and human somatic cells to pluripotency. Cell Stem Cell.

[58] Lee MR, Prasain N, Chae H-D, Kim Y-J, Mantel C, Yoder MC, Broxmeyer HE (2013). Epigenetic regulation of NANOG by miR-302 cluster-MBD2 completes induced pluripotent stem cell reprogramming. Stem Cells.

[59] Jayawardena TM, Egemnazarov B, Finch EA, Zhang L, Payne JA, Pandya K (2012). MicroRNA-mediated in vitro and in vivo direct reprogramming of cardiac fibroblasts to cardiomyocytes. Circ Res.

[60] Piubelli C, Meraviglia V, Pompilio G, D'Alessandra Y, Colombo GI, Rossini A (2014). microRNAs and cardiac cell fate. Cells.

[61] Callis TE, Pandya K, Seok HY, Tang R-H, Tatsuguchi M, Huang Z-P (2009). MicroRNA-208a is a regulator of cardiac hypertrophy and conduction in mice. J Clin Investig.

[62] Li X, Wang J, Jia Z, Cui Q, Zhang C, Wang W (2013). MiR-499 regulates cell proliferation and apoptosis during late-stage cardiac differentiation via Sox6 and cyclin D1. PLoS ONE.

[63] Yoo AS, Sun AX, Li L, Shcheglovitov A, Portmann T, Li Y (2011). MicroRNA-mediated conversion of human fibroblasts to neurons. Nature.

[64] Le C, Ran FA, Cox D, Lin S, Barretto R, Habib N (2013). Multiplex genome engineering using CRISPR/Cas systems. Science.

[65] Mali P, Yang L, Esvelt KM, Aach J, Guell M, DiCarlo JE (2013). RNA-guided human genome engineering via Cas9. Science.

[66] Nuñez JK, Chen J, Pommier GC, Cogan JZ, Replogle JM, Adriaens C (2021). Genome-wide programmable transcriptional memory by CRISPR-based epigenome editing. Cell.

[67] Weltner J, Balboa D, Katayama S, Bespalov M, Krjutškov K, Jouhilahti E-M (2018). Human pluripotent reprogramming with CRISPR activators. Nat Commun.

[68] Liu XS, Wu H, Ji X, Stelzer Y, Wu X, Czauderna S (2016). Editing DNA Methylation in the Mammalian Genome. Cell.

[69] Baumann V, Wiesbeck M, Breunig CT, Braun JM, Köferle A, Ninkovic J (2019). Targeted removal of epigenetic barriers during transcriptional reprogramming. Nat Commun.

[70] Black JB, Adler AF, Wang H-G, D'Ippolito AM, Hutchinson HA, Reddy TE (2016). Targeted epigenetic remodeling of endogenous loci by CRISPR/Cas9-based transcriptional activators directly converts fibroblasts to neuronal cells. Cell Stem Cell.

[71] Huangfu D, Maehr R, Guo W, Eijkelenboom A, Snitow M, Chen AE, Melton DA (2008). Induction of pluripotent stem cells by defined factors is greatly improved by small-molecule compounds. Nat Biotechnol.

[72] Mikkelsen TS, Hanna J, Zhang X, Ku M, Wernig M, Schorderet P (2008). Dissecting direct reprogramming through integrative genomic analysis. Nature.

[73] Shi Y, Desponts C, Do JT, Hahm HS, Schöler HR, Ding S (2008). Induction of pluripotent stem cells from mouse embryonic fibroblasts by Oct4 and Klf4 with small-molecule compounds. Cell Stem Cell.

[74] Zhou H, Wu S, Joo JY, Zhu S, Han DW, Lin T (2009). Generation of induced pluripotent stem cells using recombinant proteins. Cell Stem Cell.

[75] Mali P, Chou B-K, Yen J, Ye Z, Zou J, Dowey S (2010). Butyrate greatly enhances derivation of human induced pluripotent stem cells by promoting epigenetic remodeling and the expression of pluripotency-associated genes. Stem Cells.

[76] Zhu S, Li W, Zhou H, Wei W, Ambasudhan R, Lin T (2010). Reprogramming of human primary somatic cells by OCT4 and chemical compounds. Cell Stem Cell.

[77] Hu W, Qiu B, Guan W, Wang Q, Wang M, Li W (2015). Direct Conversion of normal and Alzheimer's disease human fibroblasts into neuronal cells by small molecules. Cell Stem Cell.

[78] Fu Y, Huang C, Xu X, Gu H, Ye Y, Jiang C (2015). Direct reprogramming of mouse fibroblasts into cardiomyocytes with chemical cocktails. Cell Res.

[79] Zhang L, Yin J-C, Yeh H, Ma N-X, Lee G, Chen XA (2015). Small molecules efficiently reprogram human astroglial cells into functional neurons. Cell Stem Cell.

[80] Li W, Zhou H, Abujarour R, Zhu S, Young Joo J, Lin T (2009). Generation of human-induced pluripotent stem cells in the absence of exogenous Sox2. Stem Cells.

[81] Zhou H, Li W, Zhu S, Joo JY, Do JT, Xiong W (2010). Conversion of mouse epiblast stem cells to an earlier pluripotency state by small molecules. J Biol Chem.

[82] Cyranoski D (2019). Woman is first to receive cornea made from 'reprogrammed' stem cells. Nature.

[83] Cyranoski D (2018). ‘Reprogrammed’ stem cells implanted into patient with Parkinson’s disease. Nature.

[84] Cyranoski D (2018). 'Reprogrammed' stem cells approved to mend human hearts for the first time. Nature.

[85] Nagoshi N, Tsuji O, Nakamura M, Okano H (2019). Cell therapy for spinal cord injury using induced pluripotent stem cells. Regen Ther.

[86] Karantzali E, Schulz H, Hummel O, Hubner N, Hatzopoulos A, Kretsovali A (2008). Histone deacetylase inhibition accelerates the early events of stem cell differentiation: transcriptomic and epigenetic analysis. Genome Biol.

[87] Kupatt C, Windisch A, Moretti A, Wolf E, Wurst W, Walter MC (2021). Genome editing for Duchenne muscular dystrophy: A glimpse of the future?. Gene Ther.

[88] Ohnuki M, Takahashi K (2015). Present and future challenges of induced pluripotent stem cells. Philos Trans R Soc Lond B Biol Sci.

[89] Wu C, Dunbar CE (2011). Stem cell gene therapy: the risks of insertional mutagenesis and approaches to minimize genotoxicity. Front Med.

[90] Eguchi T, Kuboki T (2016). Cellular reprogramming using defined factors and MicroRNAs. Stem Cells Int.

[91] Moradi S, Asgari S, Baharvand H (2014). Concise review: harmonies played by microRNAs in cell fate reprogramming. Stem Cells.

[92] Hochedlinger K, Yamada Y, Beard C, Jaenisch R (2005). Ectopic expression of Oct-4 blocks progenitor-cell differentiation and causes dysplasia in epithelial tissues. Cell.

[93] Ghaleb AM, Yang VW (2008). The pathobiology of Krüppel-like factors in colorectal cancer. Curr Colorectal Cancer Rep.

[94] Park ET, Gum JR, Kakar S, Kwon SW, Deng G, Kim YS (2008). Aberrant expression of SOX2 upregulates MUC5AC gastric foveolar mucin in mucinous cancers of the colorectum and related lesions. Int J Cancer.

[95] Qiao Y, Agboola OS, Hu X, Wu Y, Lei L (2020). Tumorigenic and immunogenic properties of induced pluripotent stem cells: a promising cancer vaccine. Stem Cell Rev Rep.

[96] de Almeida PE, Ransohoff JD, Nahid A, Wu JC (2013). Immunogenicity of pluripotent stem cells and their derivatives. Circ Res.

[97] Singh VK, Kalsan M, Kumar N, Saini A, Chandra R (2015). Induced pluripotent stem cells: applications in regenerative medicine, disease modeling, and drug discovery. Front Cell Dev Biol.

[98] Takahashi K, Yamanaka S (2013). Induced pluripotent stem cells in medicine and biology. Development.

[99] Wang H, Yang Y, Liu J, Qian L (2021). Direct cell reprogramming: approaches, mechanisms and progress. Nat Rev Mol Cell Biol.

[100] Consalvi S, Sandoná M, Saccone V (2016). Epigenetic reprogramming of muscle progenitors: inspiration for clinical therapies. Stem Cells Int.

[101] Doi D, Magotani H, Kikuchi T, Ikeda M, Hiramatsu S, Yoshida K (2020). Pre-clinical study of induced pluripotent stem cell-derived dopaminergic progenitor cells for Parkinson's disease. Nat Commun.

[102] Danisovic L, Culenova M, Csobonyeiova M (2018). Induced pluripotent stem cells for duchenne muscular dystrophy modeling and therapy. Cells.

[103] Inoue M, Kajiwara K, Yamaguchi A, Kiyono T, Samura O, Akutsu H (2019). Autonomous trisomic rescue of down syndrome cells. Lab Investig.

[104] Liu G-H, Suzuki K, Li M, Qu J, Montserrat N, Tarantino C (2014). Modelling Fanconi anemia pathogenesis and therapeutics using integration-free patient-derived iPSCs. Nat Commun.

[105] Fatima A, Gutiérrez-Garcia R, Vilchez D (2019). Induced pluripotent stem cells from Huntington's disease patients: a promising approach to define and correct disease-related alterations. Neural Regen Res.

[106] Pandelakis M, Delgado E, Ebrahimkhani MR (2020). CRISPR-based synthetic transcription factors in vivo: the future of therapeutic cellular programming. Cell Syst.

[107] Guerrero-Ramirez G-I, Valdez-Cordoba C-M, Islas-Cisneros J-F, Trevino V (2018). Computational approaches for predicting key transcription factors in targeted cell reprogramming (Review). Mol Med Rep.

[108] Fu L, Zhu X, Yi F, Liu G-H, Izpisua Belmonte JC (2014). Regenerative medicine: transdifferentiation in vivo. Cell Res.

[109] Grath A, Dai G (2019). Direct cell reprogramming for tissue engineering and regenerative medicine. J Biol Eng.

[110] Hybiak J, Jankowska K, Machaj F, Rosik J, Broniarek I, Żyluk A (2020). Reprogramming and transdifferentiation—two key processes for regenerative medicine. Eur J Pharmacol.

[111] Qin H, Zhao A, Fu X (2017). Small molecules for reprogramming and transdifferentiation. Cell Mol Life Sci.

[112] Xie X, Fu Y, Liu J (2017). Chemical reprogramming and transdifferentiation. Curr Opin Genet Dev.

[113] Tan Y, Ooi S, Wang L (2014). Immunogenicity and tumorigenicity of pluripotent stem cells and their derivatives: genetic and epigenetic perspectives. Curr Stem Cell Res Ther.

[114] Takei Y, Morioka M, Yamashita A, Kobayashi T, Shima N, Tsumaki N (2020). Quality assessment tests for tumorigenicity of human iPS cell-derived cartilage. Sci Rep.

[115] Deng X-Y, Wang H, Wang T, Fang X-T, Zou L-L, Li Z-Y, Liu C-B (2015). Non-viral methods for generating integration-free, induced pluripotent stem cells. Curr Stem Cell Res Ther.

[116] Kim Y, Jeong J, Choi D (2020). Small-molecule-mediated reprogramming: a silver lining for regenerative medicine. Exp Mol Med.

[117] Kim K, Doi A, Wen B, Ng K, Zhao R, Cahan P (2010). Epigenetic memory in induced pluripotent stem cells. Nature.

[118] Yang J, Yamato M, Nishida K, Ohki T, Kanzaki M, Sekine H (2006). Cell delivery in regenerative medicine: the cell sheet engineering approach. J Control Release.

[119] Ito N, Kii I, Shimizu N, Tanaka H, Takeda S (2017). Direct reprogramming of fibroblasts into skeletal muscle progenitor cells by transcription factors enriched in undifferentiated subpopulation of satellite cells. Sci Rep.

[120] Strumpf D, Mao C-A, Yamanaka Y, Ralston A, Chawengsaksophak K, Beck F, Rossant J (2005). Cdx2 is required for correct cell fate specification and differentiation of trophectoderm in the mouse blastocyst. Development.

[121] Guo Z, Zhang L, Wu Z, Chen Y, Wang F, Chen G (2014). In vivo direct reprogramming of reactive glial cells into functional neurons after brain injury and in an Alzheimer's disease model. Cell Stem Cell.

[122] Karow M, Camp JG, Falk S, Gerber T, Pataskar A, Gac-Santel M (2018). Direct pericyte-to-neuron reprogramming via unfolding of a neural stem cell-like program. Nat Neurosci.

[123] Ahfeldt T, Schinzel RT, Lee Y-K, Hendrickson D, Kaplan A, Lum DH (2012). Programming human pluripotent stem cells into white and brown adipocytes. Nat Cell Biol.

[124] Yamamoto K, Kishida T, Sato Y, Nishioka K, Ejima A, Fujiwara H (2015). Direct conversion of human fibroblasts into functional osteoblasts by defined factors. Proc Natl Acad Sci USA.

